# Atypical Femoral Fracture During Long-Term Treatment With Denosumab in Patients With Metastatic Bone Tumors

**DOI:** 10.7759/cureus.85052

**Published:** 2025-05-29

**Authors:** Yuka Aimono, Tomiko Sunaga, Ryo Yonezawa, Ayako Tsuboya, Mari Kogo, Ako Itoh

**Affiliations:** 1 Department of Pharmacy, Hitachi General Hospital, Hitachi, JPN; 2 Clinical Epidemiology, Division of Applied Pharmaceutical Education and Research, Hoshi University, Tokyo, JPN; 3 Department of Hospital Pharmaceutics, School of Pharmacy, Showa Medical University, Tokyo, JPN; 4 Division of Pharmacotherapeutics, Department of Clinical Pharmacy, School of Pharmacy, Showa Medical University, Tokyo, JPN; 5 Department of Breast and Thyroid Surgery, Hitachi General Hospital, Hitachi, JPN

**Keywords:** adverse effect, atypical femoral fracture, bisphosphonates, breast cancer, denosumab, long-term efficacy, metastatic bone tumors, naranjo score, osteoporosis medications, zoledronic acid hydrate

## Abstract

Bone-modifying agents (BMAs) are used to treat metastatic bone tumors and are also administered to reduce bone loss caused by bone metastases. Adverse events, such as osteonecrosis of the jaw and hypocalcemia, have been associated with long-term use of bisphosphonates; however, atypical femoral fracture (AFF) is rare. We encountered a patient who received long-term treatment with denosumab (DEN) and had a history of zoledronic acid hydrate administration. Severe complications may have been prevented if the patient had consulted with an orthopedic surgeon when she developed pain in the left thigh one month before AFF. The provision of relevant information on AFF to patients starting treatment with DEN may lead to its early detection. Since it is important to disseminate rare adverse events to the public, we report the present results based on a literature review.

## Introduction

The use of bone-modifying agents to treat metastatic bone tumors is expected to reduce the frequency of severe skeletal-related events and ameliorate bone pain [1,2]. Adverse effects, such as osteonecrosis of the jaw and hypocalcemia, have been reported; however, atypical femoral fracture (AFF) is rare. The incidence of AFF caused by denosumab (DEN) in patients with metastatic bone tumors was 0.4% in a retrospective observational study [3]. AFF was reported in 2005 as a characteristic femoral fracture in osteoporotic patients receiving long-term treatment with bisphosphonates (BPs) [4]. In 2013, the American Society for Bone and Mineral Research (ASBMR) published diagnostic criteria for AFF [5]. Major features include their location in the subtrochanteric region and diaphysis of the femur, association with no or minimal trauma, transverse or short oblique configuration, and lack of comminution. BPs are drugs that act directly on osteoclasts to suppress bone resorption and DEN is an anti-RANKL antibody that suppresses the activity of osteoclasts, which break down bone. A few studies recently described the use of DEN as a single agent to treat metastatic bone tumors [6,7]. The ASBMR Task Force reported that the risk of AFF increased with the duration of treatment with BPs [8]. AFF has been associated with the stronger suppression of bone metabolic turnover and a longer bone healing time than general fractures [9], and when complete fractures occur in cancer patients, chemotherapy must also be interrupted due to surgery and rehabilitation. Although AFF is rare, it significantly reduces quality of life and the activities of daily living. An explanation of AFF to patients and the monitoring of adverse effects are essential for its prevention. We encountered a case of AFF during long-term treatment with DEN in our hospital.

## Case presentation

**Figure 1:**
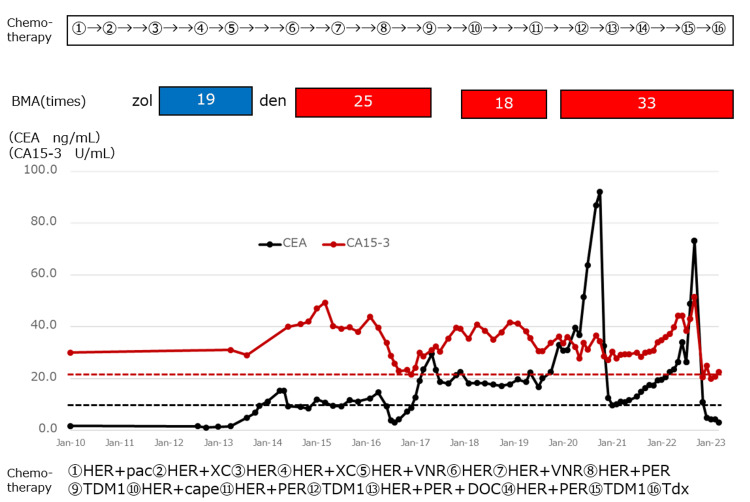
Clinical course BMAs: bone-modifying agents, DEN: denosumab, ZOL: zoledronic acid hydrate, HER: trastuzumab, pac: paclitaxel, X or Cape: capecitabine, C: cyclophosphamide, VNR: vinorelbine, PER: pertuzumab, TDM1: trastuzumab emtansine, DOC: docetaxel, TDx: trastuzumab deruxtecan, CEA: carcinoembryonic antigen, CA15-3: cancer antigen 15-3

A 71-year-old woman who was already diagnosed with right breast cancer T3N1M0IIIA (ER±, PgR-, HER3+) at the age of 51 years and underwent breast-conserving resection. Four courses of cyclophosphamide, methotrexate, and fluorouracil were administered, and the patient completed two years of treatment with anastrozole. Liver metastases appeared at the age of 56 years, and chemotherapy and molecularly targeted drugs were continued. Three years later, the patient received 19 doses of zoledronic acid hydrate (ZOL) (one year) for L3 metastases in addition to these treatments. One year later, she was switched to DEN for L3, Th8, and left scapular metastases. The patient received a total of 76 doses (8.5 years) with two breaks for tooth extraction and the stabilization of lactate dehydrogenase (LDH) and alkaline phosphatase (ALP) (Figure 1). One month before the onset of AFF, the patient began to have increasing pain in the left thigh and claudication. Since the exacerbation of bone metastasis was suspected, she was scheduled for MRI, but was injured when she tripped over a hose and fell at home. Therefore, she was urgently admitted on the same day and underwent surgery the next day. Blood and biochemical tests showed no abnormalities (Table 1). 

**Table 1:** Laboratory findings of the patient. Hb: hemoglobin, PLT: platelet count, Alb: Albumin, BUN: blood urea nitrogen, CRE: creatinine, ALP: alkaline phosphatase, CRP: C-reactive protein, TRACP-5b: tartrate-resistant acid phosphatase 5b, P1NP: procollagen type 1 N-terminal propeptide, 25(OH)VD: 25-hydroxyvitamin D

Investigation	Reference values	Units	On admission
WBC	3,500–9,000	/μL	9,500
RBC	380–500	10^4^/μL	306
Hb	11.5–11.6	g/dL	9.8
PLT	12.5–37.0	10^4^/μL	7.5
Alb	3.4–4.7	g/dL	3.3
BUN	6.0–20.0	mg/dL	29.7
CRE	0.5–0.9	mg/dL	0.65
ALP	38–113	U/L	116
Ca	8.4–11.0	mg/dL	9.4
CRP	0.0–0.3	mg/dL	0.13
TRACP-5b	120–420	mU/dL	128
P1NP	26.4–98.2	ng/mL	70
25(OH)VD	21	ng/mL	21.8

**Figure 2:**
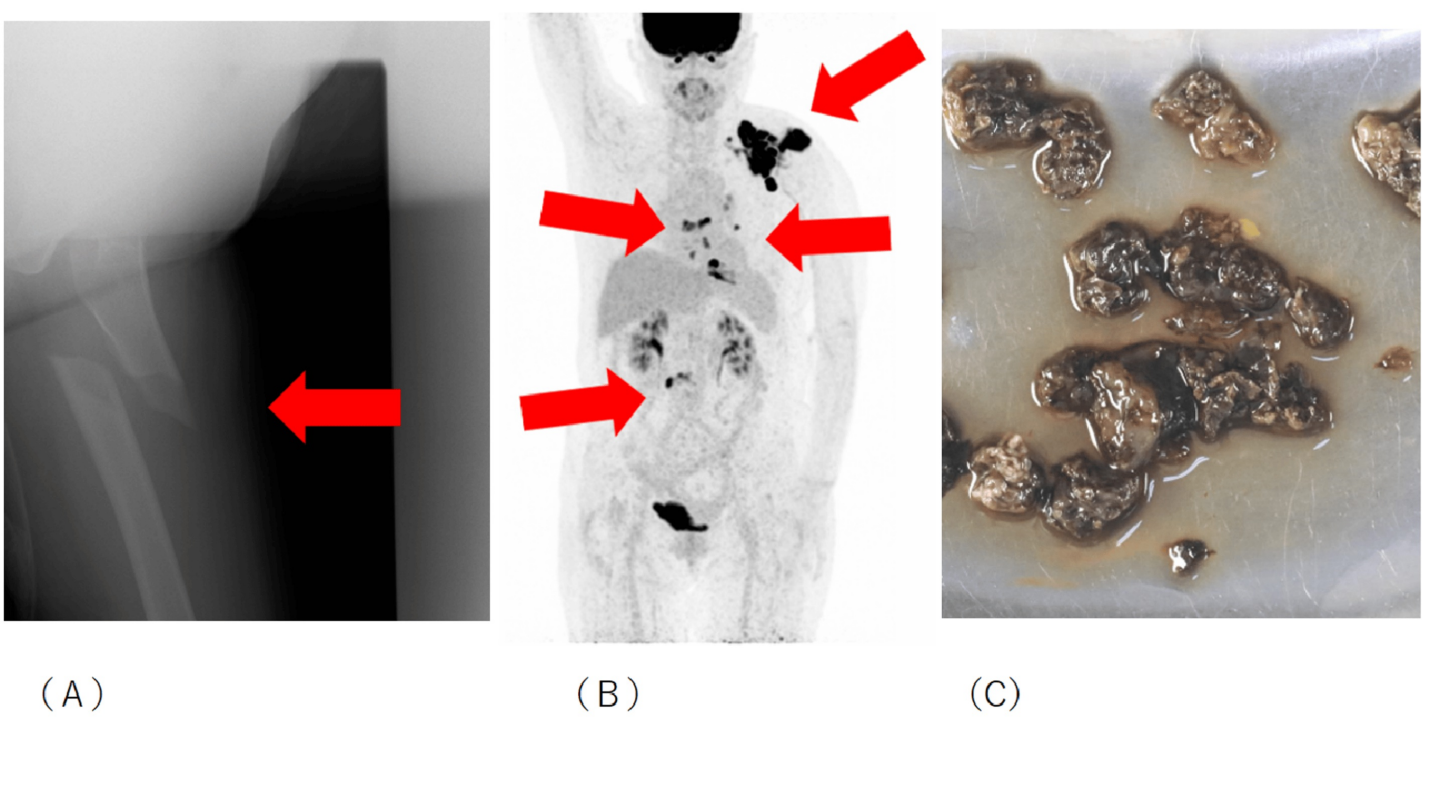
(A) X-ray at the time of injury; (B) PET, seven months before the injury; (C) Histopathology (A) Left femoral subtrochanteric fracture. (B) Positron emission tomography (PET) showed no accumulation in the left femur. (C) Necrosis and inflammation were observed, and were potentially caused by the fracture; however, there were no malignant findings, such as cancer metastases.

Previous history is ongoing chemotherapy for right breast cancer, 16th-line trastuzumab deruxtecan (TDx). Regular medications were none. X-ray at the time of injury showed a left femoral subtrochanteric fracture (Figure 2A). Seven months before the injury, positron emission tomography (PET) showed no accumulation in the left femur. Metastatic activity in the left scapula progressed slowly, while that in the thoracic spine, left ribs, and lumbar spine was rapid (Figure 2B). Under general anesthesia, the patient underwent an open reduction internal fixation (intramedullary nail fixation). The intramedullary and proximal fragment outer cortical bone at the fracture site was increased with granulation-like tissue. The operating time was 3 hours 59 minutes and blood loss was 700 mL. Curettage material from bone marrow in the left lower limb fracture site contained colloidal marrow. Bone marrow tissue showed no significant changes. There was evidence of mild inflammation and tissue necrosis, but not of vasculitis. There were also findings of necrosis and inflammation, possibly due to the fracture. There were no malignant findings, such as cancer metastases (Figure 2C).

An X-ray taken eight weeks after surgery showed bone union. All four drugs administered in the present case were examined for their potential to induce adverse drug reactions. The Naranjo Score was developed to standardize the assessment of causality for all adverse drug reactions and establishes a causal association between a drug and an adverse event [10]. It consists of 10 questions that are answered as “Yes”, “No”, or “Do not know”. Different point values (−1, 0, +1, or +2) are assigned to each answer. Total scores range from −4 to +13; the reaction is considered definite for a score ≥9, probable for 5-8, possible for 1-4, and doubtful for ≤0. All four drugs administered in the present case were examined for their potential to induce adverse drug reactions. The DEN score was 7, TDx was 4, while palonosetron (palo), and dexamethasone (dex) scores were 1 each (Table 2). Blood and biochemical tests showed no abnormalities. However, imaging, bone density, osteoporosis tests and the results of the pathological examination showed no malignant findings, DEN was discontinued because the orthopedic surgeon diagnosed AFF due to DEN with four items and subitems in the AFF diagnostic criteria [5]​​​​​ and requested the breast surgeon to discontinue DEN. The patient was wheelchair-bound on the fourth post-operative day and was moved to a rehabilitation ward for recovery four weeks post-operatively. At seven months post-operatively, the patient was able to walk with a T-cane without implant failure. This case was conducted in compliance with the Ethical Guidelines for Medical Research Involving Human Subjects and was approved by the Ethics Committee of Hitachi General Hospital (2021-55).

**Table 2:** Naranjo scores applied to the present case. The denosumab (DEN) score was 7, trastuzumab deruxtecan (TDx) was 4, while palonosetron (palo) and dexamethasone (dex) scores were 1 each.

Question	Yes	No	Do not know	Denosumab	Trastuzumab deruxtecan	Palonosetron	Dexamethasone
1. Are there previous conclusive reports on this reaction?	1	0	0	1	0	0	1
2. Did the adverse event appear after the suspect drug was administered?	2	-1	0	2	2	-1	-1
3. Did the adverse reaction improve when the drug was discontinued or a specific antagonist was administered?	1	0	0	1	0	0	0
4. Did the adverse reaction reappear when the drug was readministered?	2	-1	0	0	-1	-1	-1
5. Are there alternative causes (other than the drug) that may on their own have caused the reaction?	-1	2	0	2	2	2	2
6. Did the reaction reappear when a placebo was given?	-1	1	0	0	0	0	0
7. Was the drug detected in the blood (or other fluids) in concentrations known to be toxic?	1	0	0	0	0	0	0
8. Was the reaction more severe when the dose was increased or less severe when the dose was decreased?	1	0	0	0	0	0	0
9. Did the patient have a similar reaction to the same or similar drugs in any previous exposure?	1	0	0	0	0	0	0
10. Was the adverse event confirmed by any objective evidence?	1	0	0	1	1	1	1
Total score				7	4	1	1
Interpretation of scores				probable	possible	possible	possible
The reaction is considered definite for a score ≥9, Probable for 5–8, Possible for 1–4, and Doubtful for ≤0.							

## Discussion

The patient was then injured while an MRI was scheduled. Since many breast cancer patients with bone metastases have a promising long-term prognosis due to advances in chemotherapy, it is important to consider the risk of AFF with the long-term use of DEN. In the future, an appropriate system needs to be established for an early diagnosis of thigh pain, including the discontinuation of the suspected drug and imaging tests, while promptly collaborating with orthopedic specialists.

In the present case, bone mineral density and vitamin D levels were in the normal range. There aren’t any studies in patients with AFF, such a bone densitometry. However, there have been studies of vitamin D deficiency in patients with AFF. It has been reported that a level of serum 25-hydroxyvitamin D of less than 16 ng/mL (40 nmol/L) (odds ratio, 3.5; 95% CI, 1.7 to 18.7; P<0.001) [11]. Further, the orthopedic surgeon clinically diagnosed AFF due to DEN; however, the drug causing AFF was re-examined. The results of X-rays, PET, and a pathological examination ruled out cancer metastasis as the cause of the fracture. The drugs administered to the patient in addition to DEN were TDx, palo, and dex. Therefore, suspect drugs were re-examined using Naranjo scores. Table 1 shows that all drugs were considered to have potential side effects with a Naranjo score of 1 or more, with the maximum score of 7 for DEN. Of the four drugs, the risk of fracture is listed as an adverse event on the package inserts of DEN and dex. However, since the purpose of dex in the present case was premedication for TDx, its direct impact was considered to be negligible.

In clinical practice, ZOL was the main treatment prior to the launch of DEN. Therefore, due to their long-term survival as a result of advances in treatment, a number of patients are expected to have a history of ZOL administration. Lockwood et al. indicated the importance of considering the potential complications associated with the use of BPs in order to accurately diagnose and treat AFF in a timely manner, even in cancer patients [12]. The FDA issued guidance in 2010, which states that patients receiving BPs or DEN (particularly those on long-term treatment for three to five years) need to be instructed to report AFF symptoms and that doctors need to regularly check for these symptoms [13]. Kaku et al. examined 529 patients with metastatic bone tumors who were treated with ZOL or DEN at a single center, and five patients (0.9%) who developed AFF had received ZOL [14].

The incidence of AFF caused by DEN in patients with metastatic bone tumors was 0.4% in a retrospective observational study [3]. However, no randomized trials or other studies have been conducted on DEN-related AFF in patients with metastatic bone tumors. Therefore, further investigations are needed on the frequency of AFF in relation to age and dosages as well as on the establishment of appropriate dosing and withdrawal periods. We suggest a reassessment of the risk of fracture in female patients with a history of osteoporosis medication or ZOL use from two years after the initiation of DEN and periodically thereafter. Furthermore, if thickening of the lateral femoral cortex is observed, regular bone resorption marker measurements and radiographic examinations may help prevent AFF.

There are two limitations that need to be addressed. There was a reporting bias and the amount of individual clinical information available was limited. Since it was not possible to confirm a causal relationship, caution is needed when referring to the present results for the monitoring of adverse events in clinical practice. If the possibility of an adverse event is suggested, a case-control study is considered necessary to test the hypothesis that a relationship exists between the drug and the adverse event.

## Conclusions

Since many breast cancer patients with bone metastases have a promising long-term prognosis due to advances in chemotherapy, it is important to consider the risk of AFF with the long-term use of DEN. AFF occurred in the present patient after 8.5 years of treatment with DEN, which was longer than previously reported. In the future, an appropriate system needs to be established for an early diagnosis of thigh pain, including the discontinuation of the suspected drug and imaging tests, while promptly collaborating with orthopedic specialists.
